# Manure management strategies are interconnected with complexity across U.S. dairy farms

**DOI:** 10.1371/journal.pone.0267731

**Published:** 2022-06-03

**Authors:** Meredith T. Niles, Serge Wiltshire, Jason Lombard, Matthew Branan, Matthew Vuolo, Rajesh Chintala, Juan Tricarico

**Affiliations:** 1 Department of Nutrition and Food Sciences & Food Systems Program, University of Vermont, Burlington, Vermont, United States of America; 2 Gund Institute for Environment, University of Vermont, Burlington, Vermont, United States of America; 3 U.S. Department of Agriculture, National Animal Health Monitoring System, Animal Plant Health Inspection Service, Veterinary Services, Riverdale, MD, United States of America; 4 Dairy Management Inc., Rosemont, IL, United States of America; University of California Davis, UNITED STATES

## Abstract

Among one of the key challenges in dairy production is the management of manure in a way that is beneficial for agricultural production, with minimal environmental and public health impacts. Manure management systems (MMS)—the entire system of handling, storage, and application of manure—are diverse in countries with developed dairy industries such as the United States, enabled by a number of different technologies. The ways in which dairy farmers manage manure is driven by varying tradeoffs, including economic, social, and environmental; however, existing research has not examined the relationships between components of MMS. Here we use data from the National Animal Health Monitoring System’s Dairy 2014 study to explore the ways in which manure handling, storage, and application are related, using a series of logistic regression models and network associations. We found significant associations between how manure is handled, stored, and applied, especially driven by the consistency of manure. For solid manure, we found highly heterogeneous systems, where farmers may have a suite of alternative manure management strategies available to them, and substitution is viable. Conversely, farms using liquid manure systems have very few substitutes in their MMS, suggesting greater investment in certain infrastructures, which are not easily changed. Such findings have important implications for shifting farmers towards management practices with minimal environmental and public health impacts, demonstrating that not all farm systems are easily changed. We highlight these results in light of current policies, which may not fully capture the relationships across the MMS, and suggest that greater financing may be necessary to shift MMS on some farms. Furthermore, we suggest that different MMS have varying tradeoffs across environmental, social, and economic aspects, which demonstrates that MMS are highly individualized to a given farm’s goals and priorities.

## Introduction

The U.S. dairy sector has recently adopted a voluntary goal to have net zero carbon emissions by 2050 while supplying nutrient-dense food to feed a growing global population [1]. Manure management across dairy farms is one critical component that can enable this goal, especially since other mitigation strategies, such as feeding tannins, herbs or oils, may affect milk production [2]. Manure management in the U.S. accounts for nearly 10% of all anthropogenic methane emissions, and dairy manure management emissions are nearly half (47.3%) of all manure management emissions [3]. Manure management is required to ensure that manure is utilized as a valuable resource with minimal impact on the environment and surrounding communities [4]. Generally dairy manure is an important resource for crop production, providing valuable local nutrients for farming [5,6]. However, manure is also the focus of concern for many environmental regulations including nutrient runoff and odor restrictions [7].

Importantly, manure management may present many challenging tradeoffs between environmental, economic, community and farmer priorities [4,8–10]. The manure management system (MMS)- the entire system of various components (handling, storage, and application), which may or may not be interchangeable—involves a balance of these aforementioned tradeoffs. For example, liquid lagoon systems use large amounts of water, and may store manure anaerobically, which can increase greenhouse gas emissions [11–13]. However, the storage capacity of a lagoon ensures that farmers can apply nutrients when agronomically and climatically appropriate. For example, when nitrogen and phosphorus can be taken up by plants and not lost to the environment, which can minimize runoff and water pollution [14]. Furthermore, varying MMS have significantly different economic and labor costs, which also affect farmer decisions [15].

As dairy farmers consider these multiple tradeoffs, they employ a variety of strategies for manure handling, storage and application. Though it may appear that MMS can be easily categorized, there are multiple combinations of methods that can employ a variety of structures and equipment [15]. Broadly speaking, MMS can include three distinct components: 1) manure handling, the method of manure removal or management in animal housing or grazing areas; 2) manure storage, the infrastructure and method for holding manure until its application; and 3) manure application and manner in which manure is land applied, typically for crop fertilization [9]. However, within these three broad categories there are many technologies, structures, and equipment, which in combination represent a suite of strategies, or a system (MMS), farmers may employ to manage manure.

Importantly, while there is significant research to document the different types of MMS and their relative impact on environmental [13,16–18] and economic[10,18–21] outcomes, there is less research examining the relationships between different MMS components to each other. Here we utilize data from the USDA’s National Animal Health Monitoring System (NAHMS) Dairy 2014 study to examine the relationships among manure handling, storage, and application strategies to better understand the complexity of MMS. This is critical as farmers consider shifting towards new innovative MMS or technologies that may require significant investment or changes across the entire MMS, which may not be fully understood yet. Furthermore, the understanding of systematic relationships in MMS across dairy farms has the potential to stimulate new innovative manure management practices to mitigate misalignment challenges in terms of costs and benefits with existing strategies, and has potential to motivate dairy farmer adoption. Moreover, the understanding of relationships in MMS currently in practice at dairy farms is a prerequisite to articulate farm-level trajectories/pathways to drive sectorial-wide scaling up of transitions towards sustainable dairy farming with enhanced economic benefits and consumer acceptance for dairy products.

## 2. Methods

### 2.1 Data sources

The statistical models described here use data from the NAHMS Dairy 2014 study. NAHMS is a non-regulatory program of the United States Department of Agriculture, Animal and Plant Health Inspection Service, Veterinary Services (USDA APHIS VS) that conducts national surveys to collect health and production information for U.S. livestock, poultry, and aquaculture.

In partnership with the USDA’s National Agricultural Statistics Service (NASS), NAHMS conducted the study in 17 states representing 80.3 percent of the U.S. milk cow inventory and 76.7 percent of operations with milk cows [22,23]. The study was conducted in two phases, though the analysis presented here focuses on data collected during Phase I of the study using the General Dairy Management Questionnaire (GDMQ). There were 1,261 complete responses from dairy farmers to this questionnaire.

See the NAHMS Dairy 2014 Report 1 [22] for additional details regarding the statistical design of the study and the NAHMS Dairy 2014 Report 4 [23] for baseline descriptive statistics pertaining to MMS.

Working alongside members of the NAHMS Dairy 2014 team, including veterinary epidemiologists and statisticians, the final working dataset used in the analysis was prepared using SAS [24] to include the original study data along with variable edits and additions specific to the MMS analysis (see Supporting information, S1 Appendix for specific details on variable edits and additions). The list of MMS-specific variables used in this analysis are given in Table 1, including cow manure handling, storage or treatment, and application methods. “Other” categories in each of the MMS components were omitted from this analysis and generally showed low representation in the population [23].

**Table 1:** Manure management components, strategies, and descriptions utilized in the study. Descriptions are derived from the NAHMS Dairy 2014 Report 4 [23].

Manure Management Component	Strategy	Manure Type	Description
Manure Handling	Pasture	Solid	Manure is not handled, although the pasture might be harrowed to break up and spread manure
	Dry Lot	Solid	Manure from a dry lot which is usually scraped using a tractor with a bucket or blade
	Gutter	Solid	Conveyor with paddles that moves manure from a trough behind the cows to another handling method or storage area
	Scraper	Solid/ Liquid/ Slurry	System used to clean cow alleyways using either a scraper blade, which is moved with a chain or cable, or a tractor equipped with a bucket or blade
	Flush	Liquid/ Slurry	System in which lagoon water is used to flush manure from alleyways. Lagoon water and manure are collected, and the solids are usually separated with mechanical or gravity systems before the wastewater is recycled and used again
	Slotted Floor	Liquid/ Slurry	Floor with perforations or slots that allows manure to fall into a collection pit below
	Bedded Pack	Solid	Manure accumulates in a pack that is frequently bedded. The pack is completely removed during cleaning
	Vacuum	Liquid/ Slurry	Equipment is used to suck slurry manure from a concrete surface and into a tank
Manure Storage or Treatment System	Manure Spreader	Solid	Short-term manure storage in equipment used to scatter manure in a field on a daily or almost daily basis.
	Deep Pit	Liquid/ Slurry	Concrete or earthen-lined pit (located below cow areas) where manure accumulates and is stored.
	Slurry Tank/Basin	Liquid/ Slurry	Storage system where liquid manure is captured in a tank
	Treatment Lagoon	Liquid/ Slurry	Structure similar to a pond where manure and other wastewater accumulates, and manure decomposes. Includes both aerated and not aerated.
	Manure Pack	Solid	Accumulated manure is stored in a pack, which is inside of a barn that is frequently bedded. The pack is completely removed during cleaning.
	Other Solid Storage	Solid	Collection of solid manure outside either in a dry lot or pen or another outside area to which cows do not have access, in a building without cattle access, or with a picket dam.
	Compost	Solid	Manure is actively composted and monitored and regularly turned/mixed to aerate.
	Methane/Biogas	Liquid/ Slurry	A method for capturing gas produced when manure is stored in an anaerobic environment.
	Solid Separator	Liquid/ Slurry	Device that physically separates liquids from manure, usually through pressure.
Manure Application	Broadcast/Solid Spreader	Solid	Manure is spread widely across a field.
	Surface Application Tank Truck	Liquid/ Slurry	Manure is applied to the surface.
	Subsurface Injection	Liquid/ Slurry	Manure is injected into the ground.
	Irrigation/Sprinkler	Liquid/ Slurry	Manure applied using an above ground liquid application system.

Each component has been categorized as primarily handling solid or primarily handling liquid or slurry manure. Solid manure usually contains more than 15 percent solids and is typically found in areas where dirt, pasture, or bedding absorb moisture from the manure. Slurry manure usually contains from 5 to 15 percent solids and is generated when there is limited or no material to absorb moisture from manure. Liquid manure contains less than 5 percent solids and is generated when wastewater or rainwater is mixed with manure. For the purposes of this report, liquid and slurry manure are combined [23].

### 2.2 Descriptive analysis

Descriptive analyses were performed using SUDAAN [25], SAS-callable survey software that allows for the proper analysis of data from complex surveys by accounting for the study design. Survey-weighted relative frequencies, along with their estimates of standard errors were computed for respondents to the questions of interest (Table 1).

**Fig 1:**
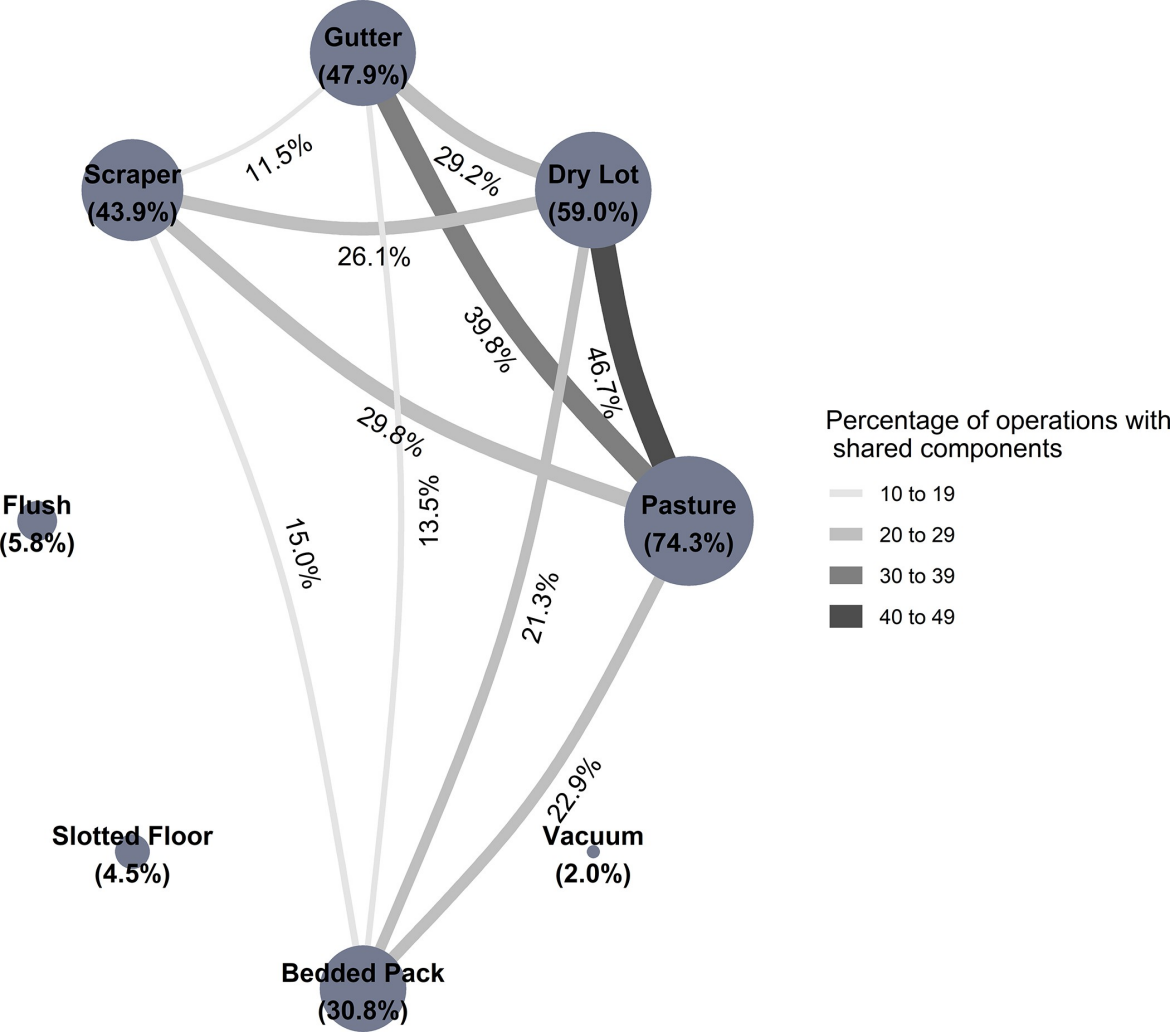
Network graph showing the percentage of operations practicing the given manure handling strategy (diameter of nodes) and the percentage of operations practicing a pair of strategies together (width of edges, with widths colored by group based on magnitude of percentage). Edges representing percentages of five or less were suppressed.

**Fig 2:**
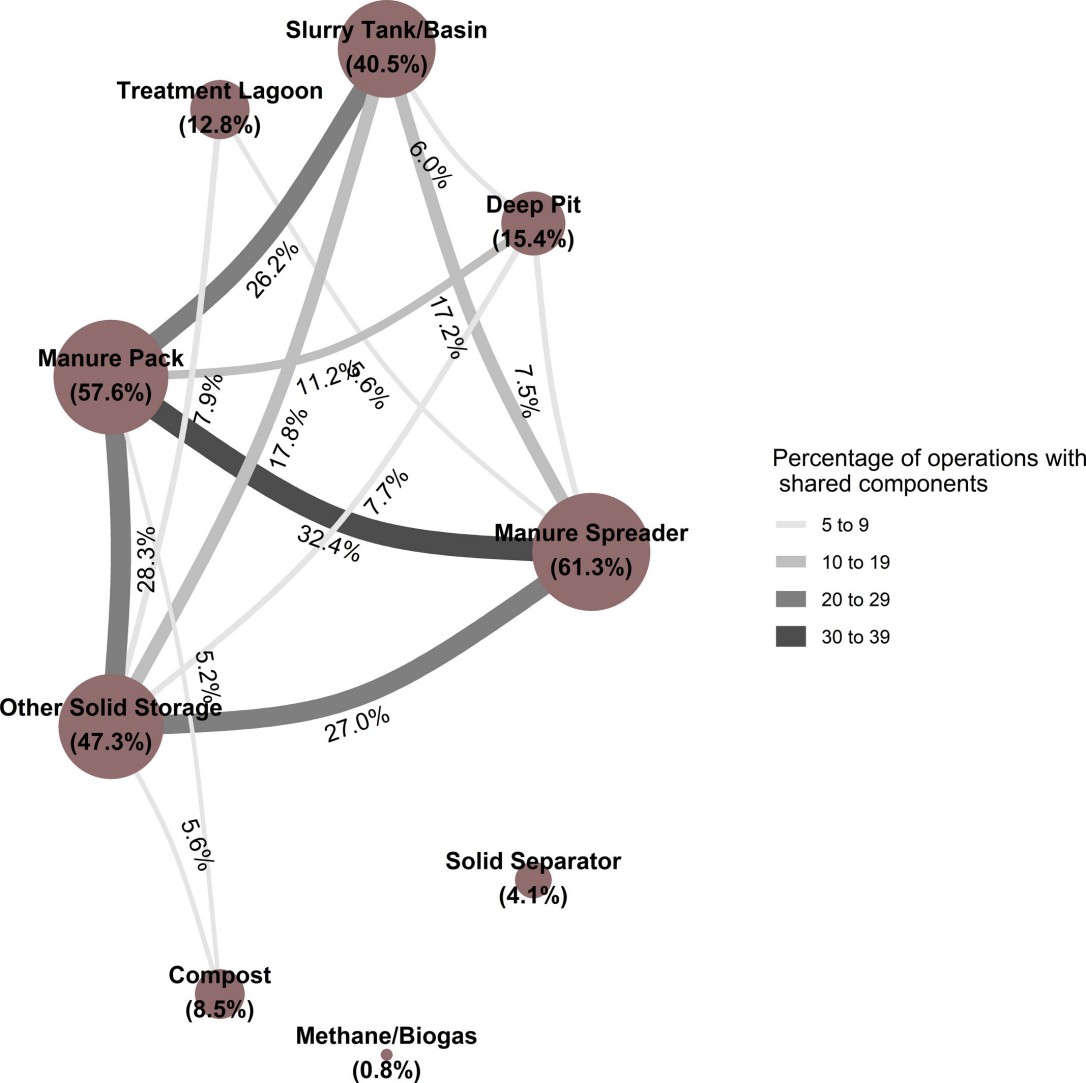
Network graph showing the percentage of operations practicing the given manure storage strategy (diameter of nodes) and the percentage of operations practicing a pair of strategies together (width of edges, with widths colored by group based on magnitude of percentage). Edges representing percentages of five or less were suppressed.

**Fig 3:**
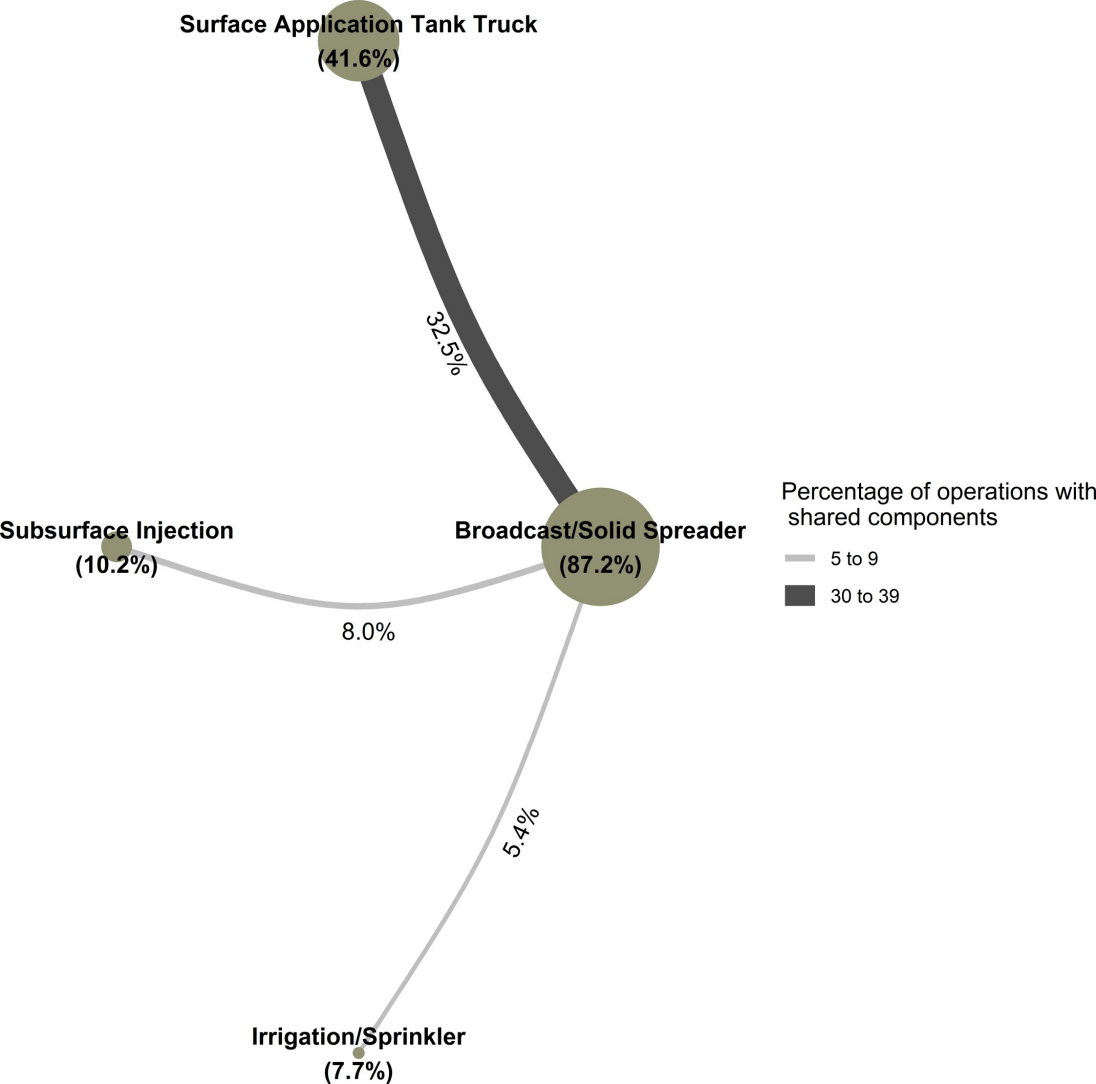
Network graph showing, for the 98.3 percent of operations that applied manure to land owned or rented by the operation, the percentage of operations practicing the given manure application strategy (diameter of nodes) and the percentage of operations practicing a pair of strategies together (width of edges, with widths colored by group based on magnitude of percentage). Edges representing percentages of five or less were suppressed.

**Fig 4:**
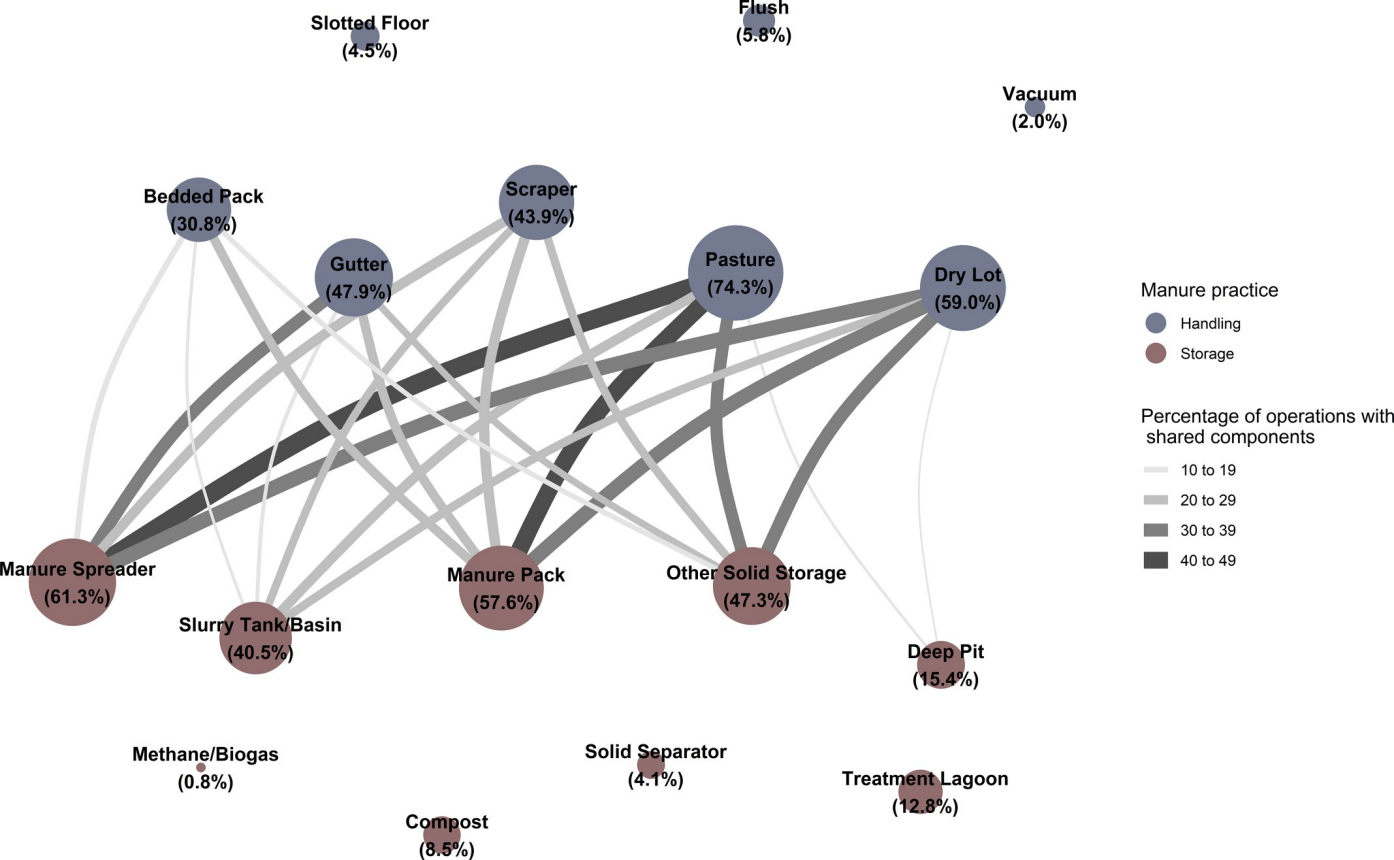
Network graph showing the percentage of operations practicing the given manure handling (blue) and storage (red) strategies (diameter of nodes) and the percentage of operations practicing handling-storage pairs of strategies together (width of edges, with widths colored by group based on magnitude of percentage). Edges representing percentages of 10 or less were suppressed Edge percentages for this figure are located in the Supporting Information S2 Appendix.

**Fig 5:**
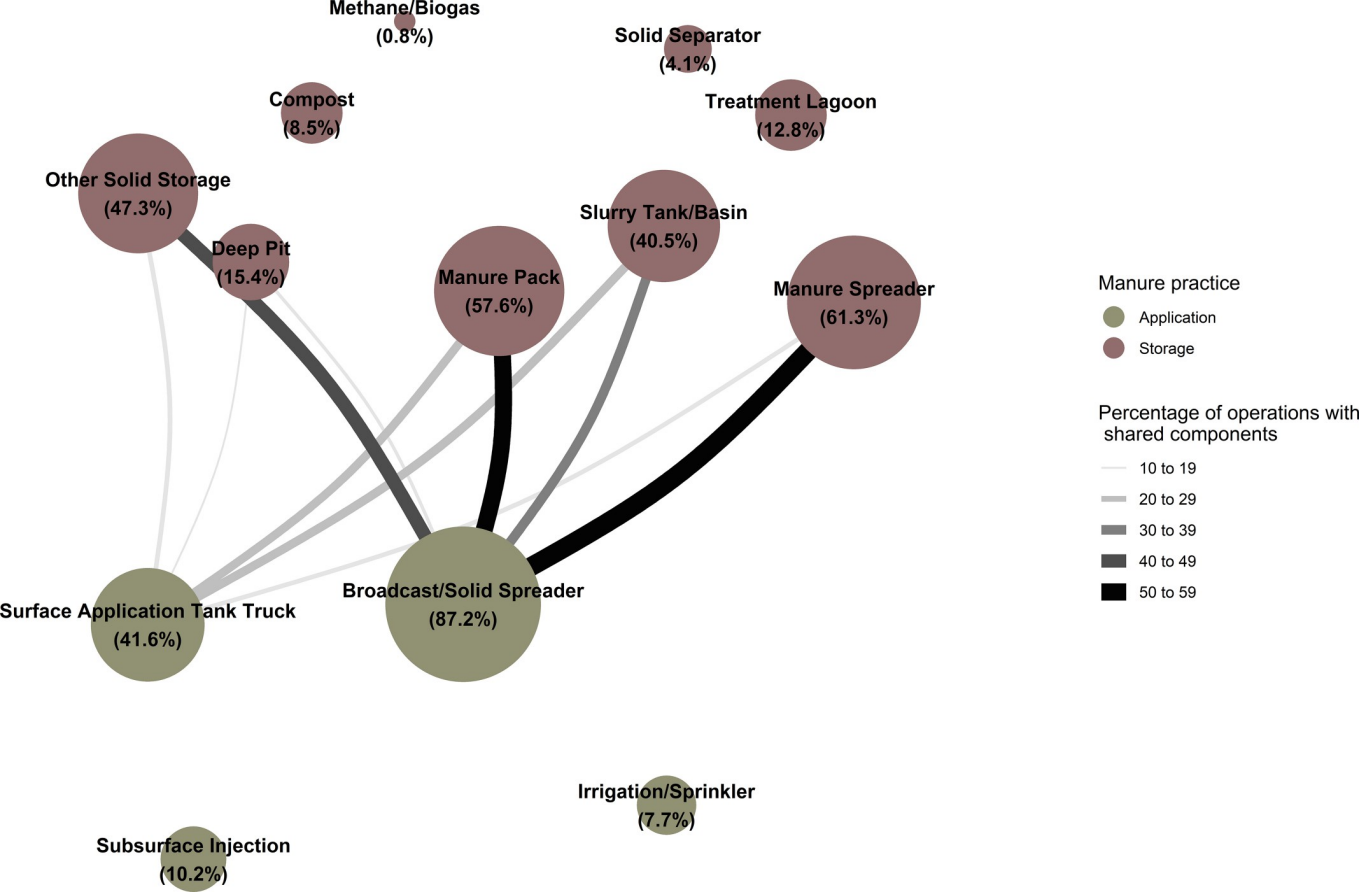
Network graph showing the percentage of operations practicing the given manure storage (red) and application (green) strategies (diameter of nodes) and the percentage of operations practicing storage-application pairs of strategies together (width of edges, with widths colored by group based on magnitude of percentage). Edges representing percentages of 10 or less were suppressed Edge percentages for this figure are located in Supporting Information S2 Appendix.

Estimates of the percentages of operations by manure handling, storage, and application method type were computed to assess the estimated relative frequency of each MMS. In addition, networks were estimated using relative frequencies using the R package *igraph* [26] and visualized using the R package *ggraph* [27] within the R statistical software [28,29]. Networks were created using estimates of percentages of operations at two levels. The first set of estimated relationships were among components of manure handling, manure storage, and manure application methods separately (Figs 1–3). The second set of estimated relationships were between components of manure handling and storage (Fig 4), and between components of manure storage and application (Fig 5).

These relationships were estimated using percentages of operations as the closeness metric to investigate the most common components of manure systems in the population. Nodes in the networks are represented by individual components and diameters of nodes are proportional to the estimated percentage of operations that had that component as a part of their MMS. Edges in the networks represent links between components and their widths are proportional to the estimated percentage of operations with the given pair of components. Component pairs representing 5 percent or less of operations for relationships among the strategies of each individual manure management component (Figs 1–3) and pairs with 10 percent of operations or less for the relationships between strategies of different components (Figs 4 and 5) were suppressed to depict the more common relationships more clearly.

### 2.3 Statistical analysis

**Fig 6:**
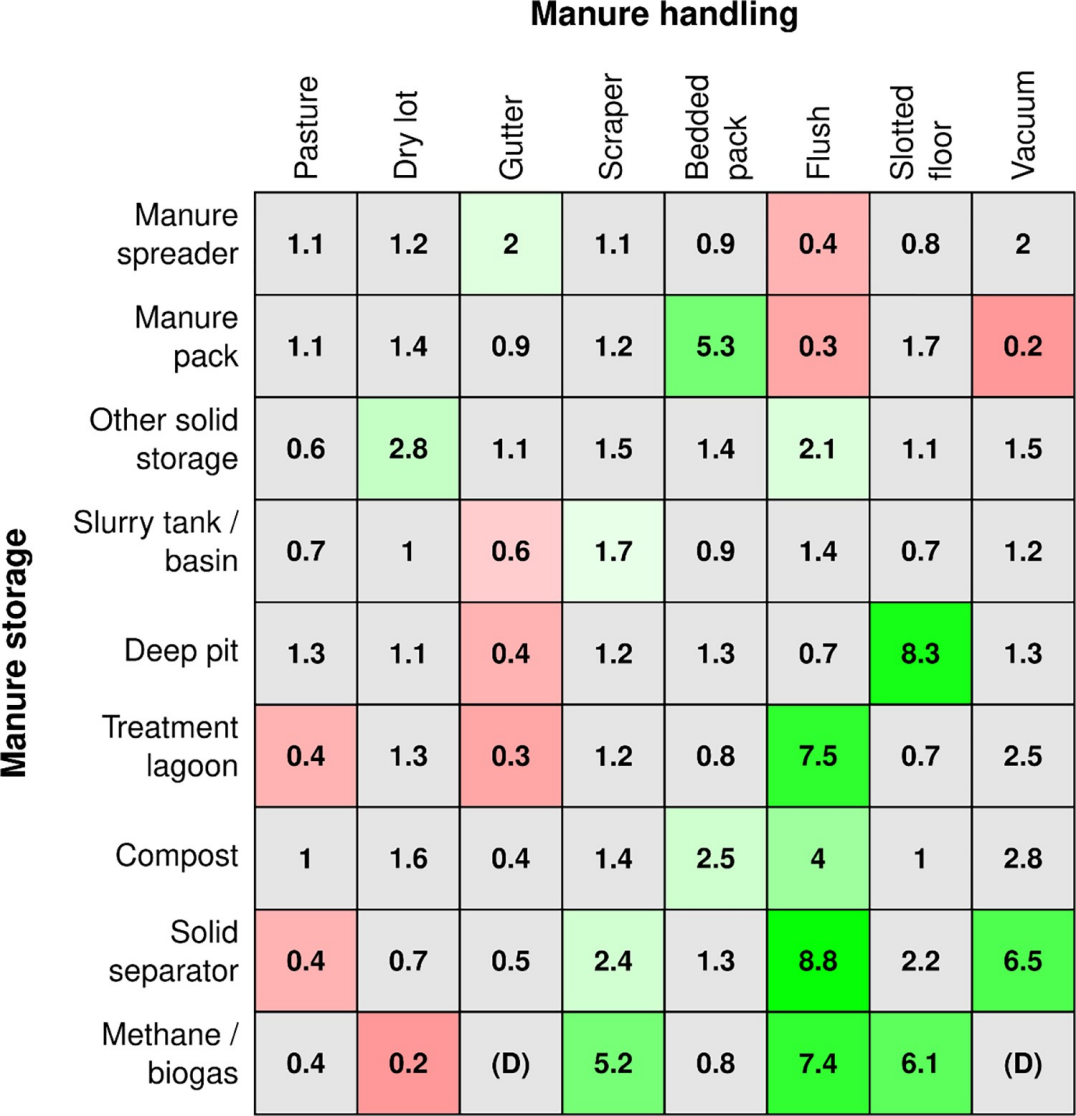
Relationship of manure handling to manure storage. Results of nine logit models taking handling methods as the independent variables, and each storage method as the dependent variable. Odds ratios (OR) from each of the logit models are given, with red shading indicating an estimated OR < 1, and green an OR > 1. Models in which the association did not achieve significance at the family-wise p < 0.05 level across rows are color-coded grey.

**Fig 7:**
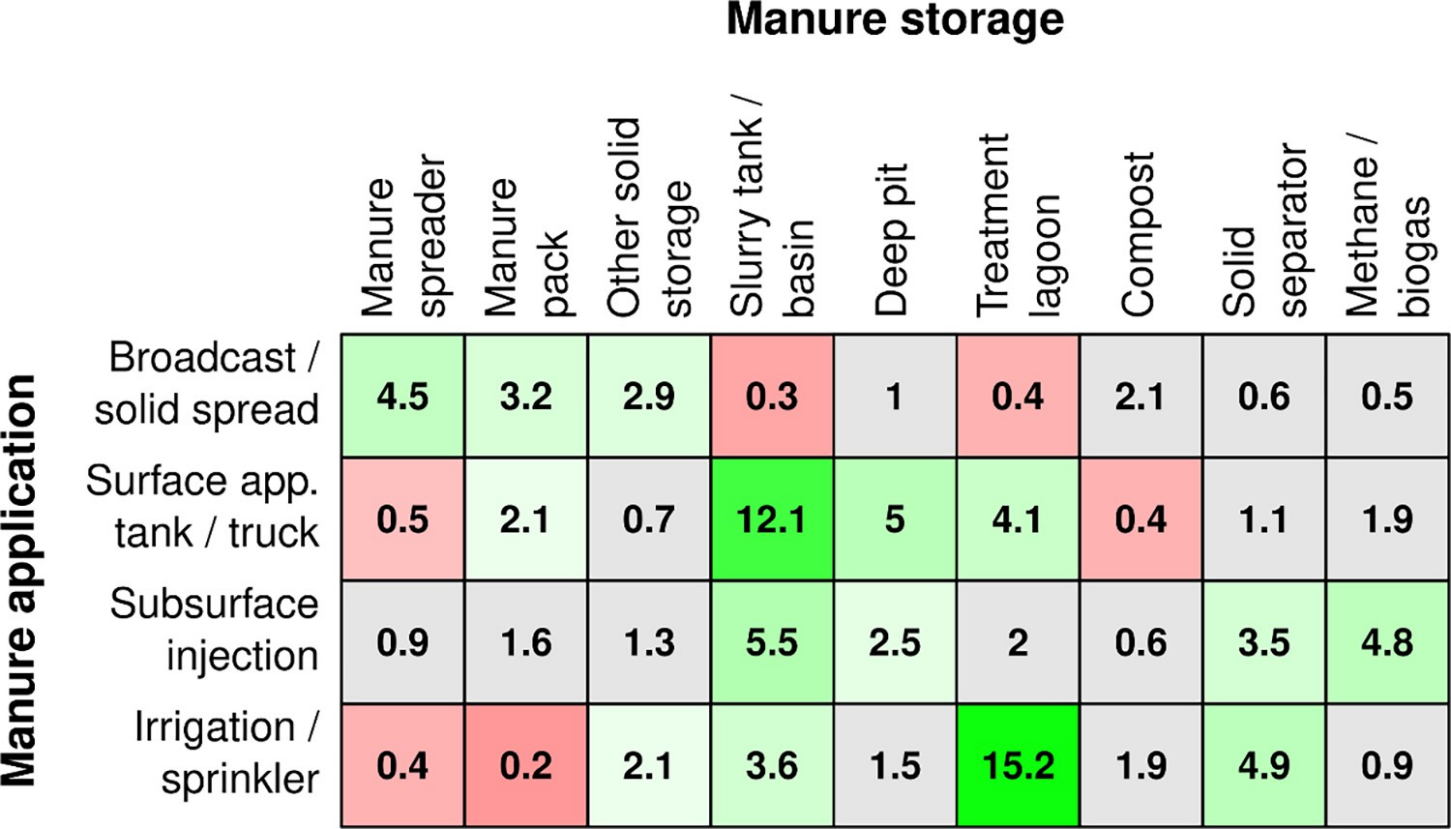
Relationship of manure application to manure storage. Results of logit models taking each storage method as the independent variable, and each application method as the dependent variable. Odds ratios (OR) from each of the logit models are given, with red shading indicating an OR < 1, and green an OR > 1. Models in which the association did not achieve significance at the family-wise p < 0.05 level across rows are color coded grey.

To assess the relationship between manure handling, storage, and application strategies, a series of logistic regression models were fit, which fall into two sets. In the first set, a single manure storage strategy was regressed on the group of eight manure handling strategies; a total of nine models were fit (Fig 6). In the second set of models, a single manure application strategy was regressed on the group of nine manure storage strategies; four models were fit (Fig 7).

In this way, we assessed the set of relationships between the three key phases of MMS as they are actually practiced on dairy operations. The results are reported as odds ratios, where ratios above one indicate a “positive association” between the two MMS components. That is, the expected odds that a dairy producer used the two strategies together is greater than the odds that the strategies were not used together. Ratios below one indicate a “negative association” between the two MMS components. That is, the expected odds that two strategies were used together is lower than the odds that the two strategies were not used together. These interpretations assume that other independent variables are held constant.

The PROC RLOGIST procedure in SUDAAN was used to run all of the logistic regression models described in this paper. In contrast to a standard logistic regression model, PROC RLOGIST incorporates the survey design and weights of the data to produce accurate point estimates and estimated standard errors. The survey-weighted logistic regression models used in this paper were modeled in the form:

logit(p)=Xβ

$$p_{ih}=P(y_{ih}=1|X_{ih}=x)$$

where *y*_*ih*_ is thebinary dependent variable (1 = the method was used; 0 = the method was not used), **X** is the design matrix, ***β*** are the regression coefficients to be estimated, *i* indexes the operation, and *h* indexes the stratum. The design matrix contains a column of 1s for the intercept, followed by columns of observed survey response data for each independent variable used in the logistic regression model, with each column corresponding to one survey variable. The design matrix has the same number of rows as there are producers represented in the underlying dataset. The independent variables were either be the 8 handling methods (Fig 6) or the 9 storage methods (Fig 7) in the two sets of models. SUDAAN estimates ***β*** by solving the weighted score equations. Standard errors are estimated using the Taylor series linearization method, assuming a without replacement design (i.e. once an operation is selected for inclusion in the sample, that same operation cannot be selected again); unequal survey weights are accounted for, and finite population corrections are applied at the stratum level. Unequal survey weights were used, as the NAHMS Dairy 2014 study design used a stratified random sample of dairy operations across State and size category strata with operations having different probabilities of selection across strata.

Statistically significant relationships between manure handling and storage methods and between storage and application methods were assessed using adjusted Wald F-test p-values [30,31] from Type III ANOVA tests [32] of significance for effects of the dependent variables. The p-value threshold to determine statistical significance were Sidak-adjusted p-value thresholds [33,34], which adjust for multiple comparisons. Per Sidak’s method, multiple comparisons were adjusted for at the model level where the threshold was calculated using the equation: $1-{\left(1-\alpha \right)}^{\frac{1}{m}}$ where *α* is the desired family-wise significance level, here chosen to be 0.05, and *m* is the number of independent variables in the regression model for which comparisons are desired. In this case, *m* = 8 and the p-value threshold equals 0.0064 for the models regressing storage methods on handling methods and *m* = 9 and the p-value threshold equals 0.0057 for the models regressing application methods on storage methods.

Statistical significance is a tool used here to focus the analysis and discussion towards common or likely relationships in the population in a complex, multivariate system of inter-related MMS components. Relationships that do not meet the significance levels are not implied to be absent or unimportant; they just did not meet the requirements to be statistically significantly associated given the methods described above and the observed data. Point estimates (and in some cases, standard error estimates) are given in order for the reader to be able to further investigate relationships beyond the binary decisions made regarding statistical significance.

## 3. Results

### 3.1 Descriptive results

The percentages of operations by manure management component strategies are presented in Table 2, along with the estimated standard errors. The estimates presented for the manure application strategies are for the 98.3 percent (SE: 0.4) of operations that applied manure to land owned or rented by the operation [23].

**Table 2:** Percentage of operations by manure handling strategy in cow housing areas, manure storage strategy, and, for the 98.3 percent of operations that applied manure to land owned or rented by the operation, percentage of operations by manure application strategy.

Manure Management Component	Strategy	Percent Operations	
		Pct.	Std. Error (SE)
Manure Handling	Pasture	74.3	(1.4)
	Dry Lot	59.0	(1.7)
	Gutter	47.9	(1.6)
	Scraper	43.9	(1.6)
	Bedded Pack	30.8	(1.6)
	Flush	5.8	(0.5)
	Slotted Floor	4.5	(0.6)
	Vacuum	2.0	(0.5)
Manure Storage	Manure Spreader	61.3	(1.6)
	Manure Pack	57.6	(1.7)
	Other Solid Storage	47.3	(1.7)
	Slurry Tank/Basin	40.5	(1.6)
	Deep Pit	15.4	(1.2)
	Any Treatment Lagoon	12.8	(0.8)
	Compost	8.5	(0.8)
	Solid Separator	4.1	(0.4)
	Methane/Biogas	0.8	(0.2)
Manure Application	Broadcast/Solid Spreader	87.2	(1.1)
	Surface Application Tank Truck	41.6	(1.6)
	Subsurface Injection	10.2	(0.8)
	Irrigation/Sprinkler	7.7	(0.6)

The most frequently practiced handling strategy was leaving manure on pasture (74.3 percent), followed by an open or dry lot that was scraped (59.0 percent), using a gutter cleaner (47.9 percent), and using an alley scraper (43.9 percent). The use of a bedded pack was used on less than a third of operations (30.8 percent).

The most frequently practiced manure storage strategies included spreading manure on a daily or nearly daily basis (61.3 percent), using a manure pack inside of a barn (57.6 percent), any of the solid storage strategies (47.3 percent), and using a slurry tank or basin (40.5 percent). By far the most frequently reported manure application strategy was use of a broadcast or solid spreader (87.2 percent) while surface application by tank wagon or tank truck was performed on 41.6 percent of operations.

More than one manure handling method and more than one storage method were used by 86.1 percent (SE: 1.3) and 81.4 percent (SE: 1.4) of operations, respectively. Also, of the 98.3 percent of operations that applied manure to land owned or rented by the operation, 42.2 percent (SE: 1.6) of operations used more than one manure application method.

The relationships among strategies within each manure management component are presented in Figs 1–3. The most common combinations of manure handling strategies (Fig 1): were

- Pasture and dry lot (46.7 percent, SE: 1.7),
- Pasture and a gutter cleaner (39.8 percent, SE: 1.7),
- Pasture and an alley scraper (29.8 percent, SE: 1.5),
- Dry lot and a gutter cleaner (29.2 percent, SE: 1.6), and
- Dry lot and an alley scraper (26.1 percent, SE: 1.4).

The most common combinations of manure storage methods (Fig 2) included:

- Manure spreader and manure pack (32.4 percent, SE: 1.6),
- Manure pack and other solid storage (28.3 percent, SE: 1.6),
- Manure spreader and other solid storage (27.0 percent, SE: 1.5),
- Manure pack and slurry tank/basin (26.2 percent, SE: 1.5), and
- Other solid storage and slurry tank/basin (17.8 percent, SE: 1.2).

The most common pairs of manure application methods (Fig 3) for the 98.3 percent of operations that applied manure to land owned or rented by the operation was a broadcast or solid spreader and surface application using a tank wagon or tank truck (32.5 percent, SE: 1.6).

The most common pairs of manure handling-storage strategies (Fig 4) reported included:

- Pasture and manure spreader (47.5 percent, SE: 1.7),
- Pasture and manure pack (44.3 percent, SE: 1.7),
- Dry lot and manure spreader (39.4 percent, SE: 1.7),
- Dry lot and manure pack (38.6 percent, SE: 1.7),
- Dry lot and other solid storage (35.5 percent, SE: 1.6),
- Gutter and manure spreader (33.8 percent, SE: 1.6),
- Pasture and other solid storage (33.4 percent, SE: 1.7).

The most common pairs of manure storage-application strategies (Fig 5) reported included: broadcast or solid spreader combined with each of manure spreader (58.2 percent, SE: 1.7), manure pack (52.9 percent, SE: 1.7), other solid storage (43.3 percent, SE: 1.7), and a slurry tank or basin (31.8 percent, SE: 1.6).

### 3.2 Statistical analysis results

#### 3.2.a Handling vs. Storage

We found both positive and negative associations between manure handling techniques and storage (Fig 6). Farms had greater odds of using manure spreaders when they used gutter systems than when they did not use gutter systems and had lower odds of spreading manure when they used flushing compared to when they did not use flushing. Manure packs are positively associated with the use of bedded packs and negatively associated with flush or vacuum handling. Other solid storage use is positively associated with dry lots and flushing. Slurry tanks are positively associated with scrapers and negatively associated with gutter systems, but neither association is very strong. Deep pit is positively associated with slotted floors and negatively associated with gutter systems. Treatment lagoons are positively associated with flushing but are negatively associated with the use of gutters or leaving manure on pasture. Farms using compost tended to be positively associated with bedded packs and flushing. Solid separators are especially associated with vacuum and flushing use and to a lesser degree scrapers but are negatively associated with manure left on pasture. Methane digesters are negatively associated with dry lots but very highly associated with flushing, slotted floors, and scrapers.

#### 3.2.b Storage vs. Application

The relationship of storage to the application of the manure is also significant in many cases, both positively and negatively (Fig 7). Farms have greater odds of using a broadcast/solid spreader application when they use a manure spreader, manure packs, or other solid storage than when they do not use these application methods, and farms have lower odds of using a broadcast/solid spreader application when they use slurry tanks and lagoons than when they do not use slurry tanks and lagoons. Surface application through tanks or trucks is most highly associated with slurry tanks, then deep pits, lagoons, and manure packs, while negatively associated with compost and manure spreader. Subsurface injection is most highly associated with the use of slurry tanks, methane digesters, solid separators, and deep pits. Finally, farms using irrigation and sprinkler applications are most associated with lagoons and solid separation, and to a lesser extent, slurry tanks and solid storage, while being negatively associated with manure packs and manure spreader.

### 3.3 Aggregated relationships

**Fig 8:**
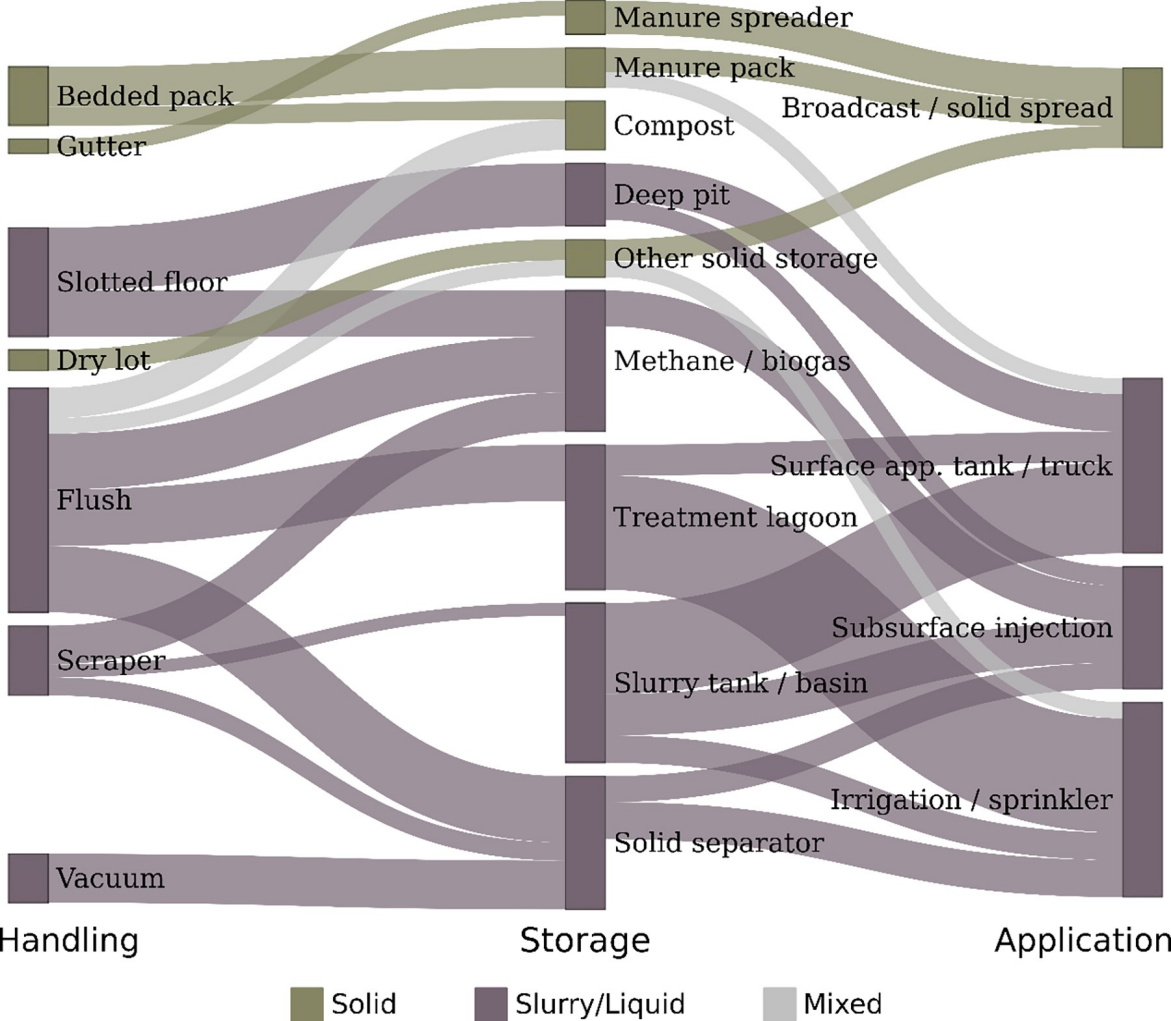
Sankey-style diagram showing connections between MMS, based on logit model results. Only models with OR > 1, and with family-wise p < 0.05 are included. Width of link increases with higher OR. Nodes and links are color coded according to their classification as elements commonly used in a solid, slurry/liquid, or mixed MMS.

Utilizing the significant odds ratios from handling, storage and application, we find clear patterns of related strategies, largely determined by the handling of manure as a liquid, slurry, or solid (Fig 8). While many of the associations in our data confirm the theoretical linkages between phases of MMS, we also find confounding results in some cases. These results suggest that farmers may often be using multiple overlapping systems, as can be observed in the NAHMS Dairy 2014 Report 4 (USDA 2018) and in Section 3.1 above. Note that the Phase I survey used in the NAHMS Dairy 2014 study did not explicitly ask detailed information on separate manure management systems within an operation, but instead focused on manure management system components used across the entire operation. Thus, sub-system analysis was not feasible given the data, and the analysis instead focused on the manure management systems used on operations, regardless if they were components in overlapping or independent systems within that operation.

As an example, operations frequently have cows in multiple housing systems, such as freestalls and dry lots where the MMS is unique to each housing system. Manure in the freestall might be removed using a flush system with a solids separator while manure in the dry lot is scraped. Thus, our results illuminate the more nuanced and complex interactions between overlapping systems that characterize MMS decision-making as it is practiced by U.S. dairy producers.

## 4. Discussion

This analysis explored the relationships between manure handling, storage and application techniques in U.S. dairy farms using nationally representative survey data. Our results highlight that there are major associations between manure handling, storage and application components, and that these relationships are reinforced by manure consistency. Liquid systems generally have a more limited set of technologies (e. g., flush and vacuum for handling, lagoon and solid separator for storage), which suggests greater standardization in liquid systems across the industry. Conversely, slurry and solid systems demonstrate greater variability across a broader range of technologies, indicating that these MMS are highly heterogeneous, and thus have a greater range of potential alternative technologies available for manure management. Previous work with these data [23] also showed that MMS systems varied across region and by size of operation, reinforcing the heterogeneity across the population of dairy farms in the U.S. due to economic, environmental and social factors. Overall, our results demonstrate that there are strong linkages between manure management components, which have significant implications for farm management and policy. We suggest three key takeaways from these results, detailed below.

First, as may seem obvious, but to our knowledge has not yet been demonstrated through empirical data across the US, the inter-related nature of these systems demonstrates how MMS are often locked in across farm systems through structural and equipment investments. For example, flush methods are significantly more likely to utilize lagoons for storage, and irrigation/sprinklers for application. This suggests that changing MMS is complex and requires not only a shift in the potential handling method, but also potential shifts in storage and application equipment as well. Flush handling can’t easily shift to scraper handling, without potentially shifting from liquid to a slurry and then investing in a surface application tank or truck system.

Manure management infrastructure can represent a significant investment, and costs can differ greatly based on MMS, region, and other production factors [15,35]. For example, data from dairy farms in Iowa suggest that the net cost to store and handle manure per cow ranges from $95.39 for two-stage sand systems to $161.56 for mattress/waterbed systems. All told, the total storage and handling costs per cow ranged from a low of $242.39 for solid systems to $349.50 for mattress/waterbed systems [15]. Furthermore, the costs of manure storage and handling are not uniform across regions; the USDA has estimated that annual manure and wastewater handling and storage costs per farm vary to be lowest in the Corn Belt and Lakes region, and highest (more than three times the cost) in the Mountain and Pacific regions [36]. These findings demonstrate that switching MMS may also be more economically viable in certain regions. Further, there is strong evidence that cost is a major factor explaining why some farmers don’t adopt manure management strategies with environmental benefits such as anaerobic digesters or manure treatment [8,37,38]. This analysis suggests that the overall costs associated with changes in single methods or technologies need to consider its cost plus the cost of related MMS components that also need to change to accommodate the new method.

Second, these results reaffirm the various tradeoffs in different MMS, including environmental, economic, and time/management tradeoffs [8,15]. For example, gutters are associated with manure spreaders, which are then highly associated with broadcast manure applications. This scenario presents several tradeoffs. The use of pasture and manure spreaders are associated with lower greenhouse gas emissions (CH_4_, N_2_O, CO_2_) and ammonia as compared with anaerobic lagoon systems [13]. However, manure spreaders require significant time and management investments [39], and are most typically used in broadcast manure applications, which are associated with increased nutrient runoff [40–42] and fecal-coliform bacteria concentration [41]. Manure spreaders, and a lack of storage capacity, could also require a farmer to apply manure in non-optimal conditions, such as before rain or on frozen ground, both of which could also lead to additional runoff and potential eutrophication [43]. Conversely, lagoon storage could contribute higher levels of greenhouse gas emissions [13], but allow the farmer flexibility for field-applying manure at optimal times to minimize nutrient runoff and water quality impacts. In this particular example, there are environmental tradeoffs in both situations depending on whether the MMS is optimizing reduction of greenhouse gas emissions or reactive nutrient runoff.

Finally, these complexities suggest that it may be challenging to shift MMS without significant infrastructure investments or consideration for tradeoffs. However, there are likely some cases in which “substitutability” may be possible, which could provide pathways for implementation of best practices for manure management. For a given farmer, these opportunities will fall within the range of possibility based on how they currently handle manure, and its resulting consistency (liquid, slurry, solid). For example, liquid handling that currently uses open lagoons could shift to covered tanks, covered lagoons or anaerobic digesters to minimize their CH_4_ and N_2_O emissions [9,44–46]. In this particular shift, it is possible that the existing manure application equipment could accommodate the new storage method, minimizing the need for new application equipment. Surface application of manure could also be changed to sub-surface injection without affecting other MMS methods; however, sub-surface injection equipment is costly and may have other tradeoffs or benefits that must be weighed.

This evidence highlights that changing MMS is not simple and requires a more nuanced exploration into the relationship of manure management components across dairy farms. Our evidence indicates that manure consistency is a critical aspect of the MMS and is not easily shifted. Furthermore, this work highlights that manure management components have varying tradeoffs, even within a single issue area such as the environment. These relationships between MMS components demonstrate that policies to assist farmers in adopting specific methods or technologies, with potential benefits for environmental or public health outcomes, might require greater financial investments than initially recognized. For example, cost share for a lagoon or methane digester may also require a farmer to invest in different handling or application equipment as well, which may not be adequately considered by current programs. This analysis also demonstrates that existing programs and policies for water quality or greenhouse gas mitigation in dairy systems may be better tailored to include practices within the range of suitability for a given dairy farm. Programs and policies could also assess the relative economic cost of manure storage and handling in certain regions to better ascertain whether existing cost-share efforts or grants are suitable for all regions, or should be adjusted. Additional economic and social science research could further map these potential cost considerations and farmer needs and priorities for manure management shifts to technologies that provide environmental or public health benefits.

## 5. Conclusion

Here we utilize representative data on U.S. dairy farms to explore the relationships of manure management components (handling, storage, and application) and the implications of such relationships for on-farm tradeoffs and management decisions. This work highlights the complexity of MMS, especially for solid and liquid/slurry systems, and demonstrates that shifts in one component of an MMS may not be possible without commensurate shifts in other components. Furthermore, we provide examples of the multiple tradeoffs that accompany MMS, with no single system having “win-win” outcomes universally. Altogether, this complexity indicates that farmer adoption of new MMS is challenging, and may involve additional tradeoffs and more economic and informational resources than have been previously considered in the literature. We encourage additional research to aid in the understanding of the social, economic, and environmental costs and tradeoffs of potential MMS changes.

## Supporting information

S1 Appendix General. (DOCX)

S2 Appendix Data for Figs 4 and 5. (DOCX)

S1 Data (CSV)
