# Supplementary material for: Manure management strategies are interconnected with complexity across U.S. dairy farms
Source: PLoS One. 2022 Jun 3;17(6):e0267731. doi: 10.1371/journal.pone.0267731 (PMC9165779; doi:10.1371/journal.pone.0267731)
Supplement: S1 Appendix — (DOCX) [file pone.0267731.s001.docx]

**Manure Management Strategies Are Interconnected with Complexity Across US Dairy Farms**

**Supporting Information, S1 Appendix**

**Meredith T. Niles^1,2^, Serge Wiltshire^1^, Jason Lombard^3^, Matthew Branan^3^, Matthew Vuolo^3^, Rajesh Chintala^4^, Juan Tricarico^4^**

1 Department of Nutrition and Food Sciences & Food Systems Program, University of Vermont

2 Gund Institute for Environment, University of Vermont

3 U.S. Department of Agriculture, National Animal Health Monitoring System, Animal Plant Health Inspection Service, Veterinary Services

4 Dairy Management Inc.

**Description of each supplementary material file:**

- “NAHMSFigure1-5Data.csv”
  - Contains data underlying the network plots for Figures 1-5.
  - Also contains data for Table 2, which can be extracted by taking all the rows where ObjectType is “Node”.
  - Column description
    - Figure: indicates the figure for which the row contains data.
    - ObjectType: indicates whether the row contains an estimate for the size of a node or for the width/color of an edge in the network graph.
    - Node1 and Node2: indicate the nodes to which the estimate corresponds. If the ObjectType is “Node” then Node1 = Node2 and the estimate gives the percentage of operations using that practice. If the ObjectType is “Edge” then Node1 is not equal to Node2 and the estimate give the percentage of operations using the combination of the two methods.
    - PercentOperations and StandardError: include the point estimate and standard error of the estimated percentage of operations using the one or combination of methods.
- “NAHMSFigure6-7Data.csv”
  - Contains data underlying the multivariate logistic regression models for Figure 6 and Figure 7.
  - Figure 8 was also created entirely from this data.
  - “(D)” estimates were suppressed for disclosure avoidance purposes
  - Identical and consecutive DependentVar values are all associated with the same logistic regression model output. For example, rows 2-10 contain the output of a single multivariate logistic regression model where “Manure Spreader” was regressed on all 8 independent variables of manure handling methods.
  - Column description
    - Figure: indicates the figure for which the row contains data.
    - DependentVar: indicates the dependent (response) variable being modeled
    - IndependentVar: indicates the independent (covariate) variable this row’s data is for
    - OR: the odds ratio point estimate given by the logistic regression model.
    - OR_CI_LB: the lower bound of the 95% confidence interval around the odds ratio point estimate.
    - OR_CI_UB: the upper bound of the 95% confidence interval around the odds ratio point estimate.
    - PValue: Type III p-value of the independent variable in this multivariate logistic regression model.
- “NAHMSFigure6-7Data.docx”
  - Table S.1 lists the variables used in the analysis as identified in the General Dairy Management Questionnaire (GDMQ) (USDA 2014).
  - Tables S.2 – S.14 contain identical data as the file “NAHMSFigure6-7Data.csv”.
