# Supplementary material for: Manure management strategies are interconnected with complexity across U.S. dairy farms
Source: PLoS One. 2022 Jun 3;17(6):e0267731. doi: 10.1371/journal.pone.0267731 (PMC9165779; doi:10.1371/journal.pone.0267731)
Supplement: S2 Appendix — (DOCX) [file pone.0267731.s002.docx]

**Manure Management Strategies Are Interconnected with Complexity Across US Dairy Farms**

**Data for Figure 6 and Figure 7**

**Meredith T. Niles^1,2^, Serge Wiltshire^1^, Jason Lombard^3^, Matthew Branan^3^, Matthew Vuolo^3^, Rajesh Chintala^4^, Juan Tricarico^4^**

1 Department of Nutrition and Food Sciences & Food Systems Program, University of Vermont

2 Gund Institute for Environment, University of Vermont

3 US Department of Agriculture, National Animal Health Monitoring System, Animal Plant Health Inspection Service

4 Dairy Management Inc.

**Variable Descriptions:**

Description of variables used in the analysis. All variables are coded as binary, with “Yes” = 1 and “No” = 3. Item codes are referencing the General Dairy Management Questionnaire (GDMQ) (USDA 2014). Some variables were recoded to take the values of multiple variables into account.

**Table S.1: Description of Variables**

| **Manure Management Component** | **Strategy** | **Item Codes Used** | **Recode (if required)** |
| --- | --- | --- | --- |
| Manure Handling | Pasture | ic790 |  |
|  | Dry Lot | ic791 |  |
|  | Gutter | ic792 |  |
|  | Scraper | ic793 |  |
|  | Flush | ic794 |  |
|  | Slotted Floor | ic795 |  |
|  | Bedded Pack | ic796 |  |
|  | Vacuum | ic797 |  |
| Manure Storage | Manure Spreader | ic827 |  |
|  | Deep Pit | ic828 |  |
|  | Slurry Tank/Basin | ic829, ic830 | “Yes” is categorized as an operation that used either a slurry tank or an earthen basin (or both).  “No” is categorized as an operation that used neither. |
|  | Treatment Lagoon | ic831, ic832 | “Yes” is categorized as an operation that used either a mechanically aerated treatment lagoon or a non-mechanically aerated treatment lagoon (or both).  “No” is categorized as an operation that used neither. |
|  | Manure Pack | ic833 |  |
|  | Other Solid Storage | ic834, ic835, ic836, ic837 | “Yes” is categorized as an operation that used at least one of the following: outside storage for solid manure not in a dry lot/pen or within a dry lot/pen, stored solid manure in a building without cattle access, or stored solid manure with picket dam.  “No” is categorized as an operation that used neither of those options. |
|  | Compost | ic838 |  |
|  | Methane/Biogas | ic839 |  |
|  | Solid Separator | ic840 |  |
| Manure Application | Broadcast/Solid Spreader | ic853 |  |
|  | Surface Application Tank Truck | ic854 |  |
|  | Subsurface Injection | ic855 |  |
|  | Irrigation/Sprinkler | ic856 |  |

**Model Outputs – Manure Storage on Manure Handling:**

Multiple logistic regression model output for the models regressing each manure storage method on the eight manure handling methods. Each variable level corresponds to one of the manure handling methods as a main effects factor in the model, plus the intercept. The point estimate for the odds ratio represents the odds ratio that a producer used the modeled manure storage method in the presence (compared to the absences) of the given handling method. The Type III p-value is the p-value testing whether the odds ratio is significantly different from 1.0. Bolded p-values are those that are statistically significant at the family-wise 0.05 significance level using Sidak-corrected p-value thresholds.

**Table S.2: “Manure Spreader” on Manure Handling Methods**

| Variable level | Odds ratio point estimate | Odds ratio 95% confidence interval | Type III p-value |
| --- | --- | --- | --- |
| Intercept | 1.0 | (0.7, 1.6) | 0.9006 |
| Pasture | 1.1 | (0.8, 1.5) | 0.5041 |
| Dry Lot | 1.2 | (0.9, 1.6) | 0.3074 |
| Gutter | **2.0** | **(1.4, 2.8)** | **0.0001** |
| Scraper | 1.1 | (0.8, 1.6) | 0.5269 |
| Flush | **0.4** | **(0.2, 0.5)** | **<0.0001** |
| Slotted Floor | 0.8 | (0.5, 1.4) | 0.4819 |
| Bedded Pack | 0.9 | (0.6, 1.2) | 0.3561 |
| Vacuum | 2.0 | (1.0, 4.0) | 0.0510 |

**Table S.3: “Deep Pit” on Manure Handling Methods**

| Variable level | Odds ratio point estimate | Odds ratio 95% confidence interval | Type III p-value |
| --- | --- | --- | --- |
| Intercept | 0.1 | (0.1, 0.2) | <0.0001 |
| Pasture | 1.3 | (0.9, 2.0) | 0.1769 |
| Dry Lot | 1.1 | (0.7, 1.8) | 0.5700 |
| Gutter | **0.4** | **(0.3, 0.7)** | **0.0019** |
| Scraper | 1.2 | (0.8, 1.9) | 0.4089 |
| Flush | 0.7 | (0.3, 1.5) | 0.3542 |
| Slotted Floor | **8.3** | **(4.7, 14.9)** | **<0.0001** |
| Bedded Pack | 1.3 | (0.9, 2.0) | 0.2286 |
| Vacuum | 1.3 | (0.3, 6.3) | 0.7153 |

**Table S.4: “Slurry Tank/Basin” on Manure Handling Methods**

| Variable level | Odds ratio point estimate | Odds ratio 95% confidence interval | Type III p-value |
| --- | --- | --- | --- |
| Intercept | 0.9 | (0.6, 1.3) | 0.5205 |
| Pasture | 0.7 | (0.5, 1.0) | 0.0525 |
| Dry Lot | 1.0 | (0.7, 1.3) | 0.8825 |
| Gutter | **0.6** | **(0.4, 0.8)** | **0.0017** |
| Scraper | **1.7** | **(1.2, 2.3)** | **0.0012** |
| Flush | 1.4 | (1.0, 2.2) | 0.0841 |
| Slotted Floor | 0.7 | (0.4, 1.3) | 0.2304 |
| Bedded Pack | 0.9 | (0.7, 1.2) | 0.4737 |
| Vacuum | 1.2 | (0.6, 2.5) | 0.6640 |

**Table S.5: “Treatment Lagoon” on Manure Handling Methods**

| Variable level | Odds ratio point estimate | Odds ratio 95% confidence interval | Type III p-value |
| --- | --- | --- | --- |
| Intercept | 0.3 | (0.2, 0.5) | <0.0001 |
| Pasture | **0.4** | **(0.3, 0.5)** | **<0.0001** |
| Dry Lot | 1.3 | (0.9, 2.1) | 0.2138 |
| Gutter | **0.3** | **(0.2, 0.5)** | **<0.0001** |
| Scraper | 1.2 | (0.7, 1.9) | 0.5213 |
| Flush | **7.5** | **(4.7, 11.9)** | **<0.0001** |
| Slotted Floor | 0.7 | (0.4, 1.2) | 0.1744 |
| Bedded Pack | 0.8 | (0.5, 1.2) | 0.2382 |
| Vacuum | 2.5 | (1.0, 6.1) | 0.0488 |

**Table S.6: “Manure Pack” on Manure Handling Methods**

| Variable level | Odds ratio point estimate | Odds ratio 95% confidence interval | Type III p-value |
| --- | --- | --- | --- |
| Intercept | 0.7 | (0.4, 1.1) | 0.0849 |
| Pasture | 1.1 | (0.8, 1.6) | 0.5624 |
| Dry Lot | 1.4 | (1.0, 1.9) | 0.0434 |
| Gutter | 0.9 | (0.6, 1.3) | 0.4872 |
| Scraper | 1.2 | (0.9, 1.7) | 0.2885 |
| Flush | **0.3** | **(0.1, 0.5)** | **<0.0001** |
| Slotted Floor | 1.7 | (0.8, 3.3) | 0.1513 |
| Bedded Pack | **5.3** | **(3.7, 7.7)** | **<0.0001** |
| Vacuum | **0.2** | **(0.1, 0.5)** | **0.0010** |

**Table S.7: “Other Solid Storage” on Manure Handling Methods**

| Variable level | Odds ratio point estimate | Odds ratio 95% confidence interval | Type III p-value |
| --- | --- | --- | --- |
| Intercept | 0.4 | (0.3, 0.7) | 0.0001 |
| Pasture | 0.6 | (0.5, 0.9) | 0.0074 |
| Dry Lot | **2.8** | **(2.0, 3.8)** | **<0.0001** |
| Gutter | 1.1 | (0.8, 1.6) | 0.4450 |
| Scraper | 1.5 | (1.1, 2.1) | 0.0147 |
| Flush | **2.1** | **(1.3, 3.3)** | **0.0017** |
| Slotted Floor | 1.1 | (0.6, 2.1) | 0.7868 |
| Bedded Pack | 1.4 | (1.0, 1.9) | 0.0469 |
| Vacuum | 1.5 | (0.6, 3.8) | 0.4130 |

**Table S.8: “Compost” on Manure Handling Methods**

| Variable level | Odds ratio point estimate | Odds ratio 95% confidence interval | Type III p-value |
| --- | --- | --- | --- |
| Intercept | 0.0 | (0.0, 0.1) | <0.0001 |
| Pasture | 1.0 | (0.7, 1.6) | 0.8654 |
| Dry Lot | 1.6 | (1.0, 2.7) | 0.0744 |
| Gutter | 0.4 | (0.2, 0.8) | 0.0126 |
| Scraper | 1.4 | (0.8, 2.4) | 0.2082 |
| Flush | **4.0** | **(2.4, 6.7)** | **<0.0001** |
| Slotted Floor | 1.0 | (0.5, 2.0) | 0.9326 |
| Bedded Pack | **2.5** | **(1.6, 4.0)** | **0.0001** |
| Vacuum | 2.8 | (1.3, 5.7) | 0.0068 |

**Table S.9: “Methane/Biogas” on Manure Handling Methods**

| Variable level | Odds ratio point estimate | Odds ratio 95% confidence interval | Type III p-value |
| --- | --- | --- | --- |
| Intercept | 0.0 | (0.0, 0.0) | <0.0001 |
| Pasture | 0.4 | (0.1, 1.2) | 0.1087 |
| Dry Lot | **0.2** | **(0.1, 0.6)** | **0.0010** |
| Gutter | (D) | (D) | (D) |
| Scraper | **5.2** | **(2.2, 12.1)** | **0.0002** |
| Flush | 7.4 | (3.2, 17.0) | **<0.0001** |
| Slotted Floor | **6.1** | **(2.5, 15.1)** | **0.0001** |
| Bedded Pack | 0.8 | (0.3, 2.1) | 0.7058 |
| Vacuum | (D) | (D) | (D) |

(D) estimates were suppressed for disclosure avoidance purposes

**Table S.10: “Solid Separator” on Manure Handling Methods**

| Variable level | Odds ratio point estimate | Odds ratio 95% confidence interval | Type III p-value |
| --- | --- | --- | --- |
| Intercept | 0.0 | (0.0, 0.1) | <0.0001 |
| Pasture | **0.4** | **(0.3, 0.7)** | **0.0002** |
| Dry Lot | 0.7 | (0.4, 1.1) | 0.1286 |
| Gutter | 0.5 | (0.2, 0.9) | 0.0217 |
| Scraper | **2.4** | **(1.4, 4.1)** | **0.0012** |
| Flush | **8.8** | **(5.3, 14.8)** | **<0.0001** |
| Slotted Floor | 2.2 | (1.1, 4.2) | 0.0190 |
| Bedded Pack | 1.3 | (0.8, 2.1) | 0.3156 |
| Vacuum | **6.5** | **(3.1, 13.4)** | **<0.0001** |

**Model Outputs – Manure Application on Manure Storage:**

Multiple logistic regression model output for the models regressing each manure application method on the nine manure storage methods. Each variable level corresponds to one of the manure storage methods as a main effects factor in the model, plus the intercept. The point estimate for the odds ratio represents the odds ratio that a producer used the modeled manure storage method in the presence (compared to the absences) of the given storage method. The Type III p-value is the p-value testing whether the odds ratio is significantly different from 1.0. Bolded p-values are those that are statistically significant at the family-wise 0.05 significance level using Sidak-corrected p-value thresholds.

**Table S.11: “Broadcast/Solid Spreader” on Manure Storage Methods**

| Variable level | Odds ratio point estimate | Odds ratio 95% confidence interval | Type III p-value |
| --- | --- | --- | --- |
| Intercept | 2.4 | (1.3, 4.3) | 0.0052 |
| Manure Spreader | **4.5** | **(2.6, 7.5)** | **<0.0001** |
| Deep Pit | 1.0 | (0.5, 1.9) | 0.9779 |
| Slurry Tank/Basin | **0.3** | **(0.2, 0.6)** | **<0.0001** |
| Treatment Lagoon | **0.4** | **(0.3, 0.8)** | **0.0046** |
| Manure Pack | **3.2** | **(2.0, 5.2)** | **<0.0001** |
| Other Solid Storage | **2.9** | **(1.7, 4.8)** | **<0.0001** |
| Compost | 2.1 | (1.2, 3.9) | 0.0130 |
| Methane/Biogas | 0.5 | (0.1, 1.7) | 0.2467 |
| Solid Separator | 0.6 | (0.3, 1.3) | 0.1603 |

**Table S.12: “Surface Application Tank/Truck” on Manure Storage Methods**

| Variable level | Odds ratio point estimate | Odds ratio 95% confidence interval | Type III p-value |
| --- | --- | --- | --- |
| Intercept | 0.2 | (0.1, 0.3) | <0.0001 |
| Manure Spreader | **0.5** | **(0.3, 0.7)** | **<0.0001** |
| Deep Pit | **5.0** | **(3.0, 8.4)** | **<0.0001** |
| Slurry Tank/Basin | **12.1** | **(8.4, 17.3)** | **<0.0001** |
| Treatment Lagoon | **4.1** | **(2.3, 7.1)** | **<0.0001** |
| Manure Pack | **2.1** | **(1.5, 2.9)** | **<0.0001** |
| Other Solid Storage | 0.7 | (0.5, 1.0) | 0.0412 |
| Compost | **0.4** | **(0.2, 0.7)** | **0.0029** |
| Methane/Biogas | 1.9 | (0.3, 11.1) | 0.4662 |
| Solid Separator | 1.1 | (0.5, 2.5) | 0.8453 |

**Table S.13: “Subsurface Injection” on Manure Storage Methods**

| Variable level | Odds ratio point estimate | Odds ratio 95% confidence interval | Type III p-value |
| --- | --- | --- | --- |
| Intercept | 0.0 | (0.0, 0.0) | <0.0001 |
| Manure Spreader | 0.9 | (0.6, 1.4) | 0.7902 |
| Deep Pit | **2.5** | **(1.6, 4.1)** | **0.0002** |
| Slurry Tank/Basin | **5.5** | **(3.5, 8.6)** | **<0.0001** |
| Treatment Lagoon | 2.0 | (1.1, 3.5) | 0.0199 |
| Manure Pack | 1.6 | (1.0, 2.5) | 0.0396 |
| Other Solid Storage | 1.3 | (0.9, 2.0) | 0.1824 |
| Compost | 0.6 | (0.3, 1.0) | 0.0624 |
| Methane/Biogas | **4.8** | **(1.6, 13.9)** | **0.0044** |
| Solid Separator | **3.5** | **(1.6, 7.5)** | **0.0020** |

**Table S.14: “Irrigation/Sprinkler” on Manure Storage Methods**

| Variable level | Odds ratio point estimate | Odds ratio 95% confidence interval | Type III p-value |
| --- | --- | --- | --- |
| Intercept | 0.0 | (0.0, 0.1) | <0.0001 |
| Manure Spreader | **0.4** | **(0.3, 0.7)** | **0.0018** |
| Deep Pit | 1.5 | (0.6, 3.5) | 0.3673 |
| Slurry Tank/Basin | **3.6** | **(2.3, 5.8)** | **<0.0001** |
| Treatment Lagoon | **15.2** | **(9.8, 23.5)** | **<0.0001** |
| Manure Pack | **0.2** | **(0.1, 0.3)** | **<0.0001** |
| Other Solid Storage | **2.1** | **(1.3, 3.6)** | **0.0035** |
| Compost | 1.9 | (1.0, 3.6) | 0.0444 |
| Methane/Biogas | 0.9 | (0.3, 3.1) | 0.8402 |
| Solid Separator | **4.9** | **(2.7, 8.9)** | **<0.0001** |
